# Effectiveness of *De Qi* during acupuncture for the treatment of tinnitus: study protocol for a randomized controlled trial

**DOI:** 10.1186/1745-6215-15-397

**Published:** 2014-10-15

**Authors:** Hui Xie, Xinrong Li, Jiaqin Lai, Yanan Zhou, Caiying Wang, Jiao Liang

**Affiliations:** Department of Otorhinolaryngology, Head and Neck Surgery of the Teaching Hospital of Chengdu University of Traditional Chinese Medicine, Chengdu, Sichuan Province 610072 PR China; Chengdu University of Traditional Chinese Medicine, Chengdu, Sichuan Province 610072 PR China

**Keywords:** Tinnitus, Acupuncture, *De Qi*, Randomized controlled trial

## Abstract

**Background:**

Acupuncture has been used in China to treat tinnitus for a long time. There is debate as to whether or not *De Qi* is a key factor in achieving the efficacy of acupuncture. However, there is no sufficient evidence obtained from randomized controlled trials to confirm the role of *De Qi* in the treatment of acupuncture for tinnitus. This study aims to identify the effect of *De Qi* for patients who receive acupuncture to alleviate tinnitus by a prospective, double-blind, randomized, sham-controlled trial.

**Methods and design:**

This study compares two acupuncture groups (with or without manipulation) in 292 patients with a history of subjective tinnitus. The trial will be conducted in the Teaching Hospital of Chengdu University of Traditional Chinese Medicine. In the study, the patients will be randomly assigned into two groups according to a computer-generated randomization list and assessed prior to treatment. Then, they will receive 5 daily sessions of 30 minutes each time for 4 consecutive weeks and undergo a 12-week follow-up phase. The administration of acupuncture follows the guidelines for clinical research on acupuncture (WHO Regional Publication, Western Pacific Series Number 15, 1995), and is performed double-blind by physicians well-trained in acupuncture. The measures of outcome include the subjective symptoms scores and quantitative sensations of *De Qi* evaluated by Visual Analog Scales (VAS) and the Chinese version of the ‘modified’ Massachusetts General Hospital Acupuncture Sensation Scale (C-MMASS). Furthermore, adverse events are recorded and analyzed. If any subjects are withdrawn from the trial, intention-to-treat analysis (ITT) and per-protocol (PP) analysis will be performed.

**Discussion:**

The key features of this trial include the randomization procedures, large sample and the standardized protocol to evaluate *De Qi* qualitatively and quantitatively in the treatment of acupuncture for tinnitus. The trial will be the first study with a high evidence level in China to assess the efficacy of *De Qi* in the treatment of tinnitus in a randomized, double-blind, sham-controlled manner.

**Trial registration:**

Chinese Clinical Trial Registry: ChiCTR-TRC-14004720 (6 May 2014).

## Background

Tinnitus is a spontaneous, internally generated noise, which can be heard in the ear or in the head. The prevalence of tinnitus is estimated among 8 to 30% [1] and the exact causes of it remain unknown. Malfunction of the auditory end-organ, which could be caused by any condition, might cause subjective tinnitus, but the commonest cause is age-related degeneration. Other causes include Meniere’s disease, trauma (acoustic or chemical), and cardiovascular diseases. The reasons for the onset of tinnitus of each individual are still unclear and this may explain why different treatments of the disease are beneficial for some patients but not for others [2]. Patients with tinnitus complain bitterly about the persistent noise and impairments of quality of life and work are common, particularly in patients with moderate/severe tinnitus.

Different treatment methods have been tried to alleviate tinnitus, which include sound therapy (masking) [3], repetitive transcranial magnetic stimulation therapy [4], cognitive behavioral therapy, tinnitus retraining therapy and pharmacological treatment (antidepressants, anxiolytics and night sedatives). Although there are so many treatment options for tinnitus patients these methods are not universally effective due to its unclear etiology and pathogenesis. Also because levels of evidence of most of the trials to treat tinnitus are generally limited, the efficacy of most interventions for tinnitus benefit remains to be demonstrated conclusively [5].

As a result, complementary or alternative therapies, such as acupuncture, are attractive to both patients and practitioners. In China, acupuncture has been used for alleviating tinnitus since ancient times. Some studies aiming to evaluate the therapeutic effects of acupuncture on the neurogenic tinnitus indicate that acupuncture can improve tinnitus in some selected patients [6]. There is also a theory stating that acupuncture may influence the function of the olivocochlear nucleus [7]. In order to achieve the best therapeutic effect from acupuncture, *De Qi* is emphasized in traditional Chinese theory [8]. Describing the compound word ‘*De Qi*’ separately, *De* means ‘obtain’ and *Qi* indicates the most primitive substance in the universe that remains in constant motion. In general, the term means a stimulation that reaches a threshold that elicits nerve impulse transmission to the cerebral cortex. In vivid description, *De Qi* describes an acupuncture needle sensation of soreness, tingling, fullness, aching, cool, warmth and heaviness, and a radiating sensation at and around the acupoints. *De Qi* has been explained to some degree to be in closely correlated with the intensity of the stimulus, so it plays a pivotal role in achieving the best therapeutic effects [9–14]. Although the concept of *De Qi* is a long-held belief that ensures the efficacy of acupuncture for tinnitus, it has not been confirmed qualitatively and quantitatively by sufficient evidence from randomized controlled trials. Additionally, without the feeling of *De Qi*, the therapeutic effect of acupuncture for tinnitus may be compromised. Thus, we designed a randomized, double-blind clinical trial to compare the efficacy of acupuncture with either strong (intended to elicit *De Qi*) or weak stimulation among patients with subjective tinnitus. The design and methodologies of this study have been approved by Sichuan Regional Ethics Review Committee on Traditional Chinese Medicine with an ethics approval number of 2014KL-006. The work reported in this article is registered with an identifier (ChiCTR-TRC-14004720) by the Chinese Clinical Trial Registry.

## Methods and design

### Study design

This is a prospective, double-blind, randomized and sham-controlled trial that compares two acupuncture groups (with or without manipulation). This study protocol has been approved by Sichuan Regional Ethics Review Committee on Traditional Chinese Medicine (project number: 2014KL-006). Also, the trial reported in this article is registered by the Chinese Clinical Trial Registry with an identifier of ChiCTR-TRC-14004720. The trial will be performed according to the principles of the Declaration of Helsinki (Version Edinburgh 2000). In addition, written informed consents will be taken from all patients.

### Population

**Figure 1:**
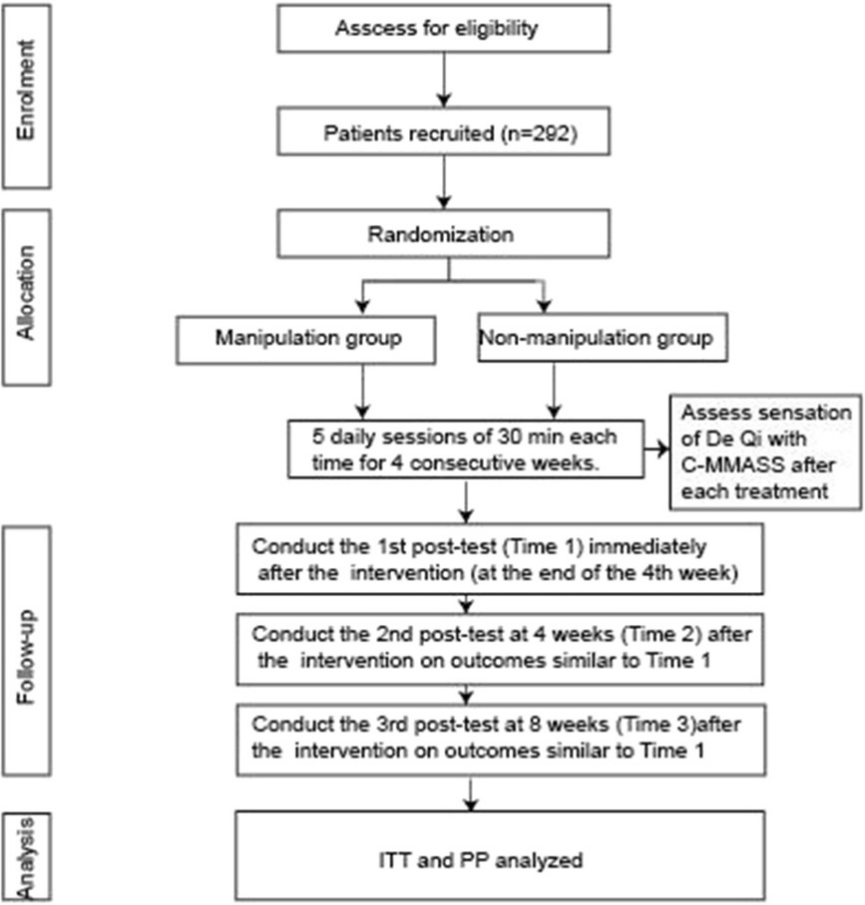
**Tools used for outcome measurement: Visual Analog Scale (VAS), Tinnitus Handicap Inventory (THI), Tinnitus Severity Index Questionnaire, Tinnitus Loudness Questionnaire, and 10-cm VAS C-MMSAA: Chinese version of the ‘modified’ Massachusetts General Hospital Acupuncture Sensation Scale (C-MMASS).** A flowchart of the procedure of this clinical trial.

The study population will be recruited at the Teaching Hospital of Chengdu University of Traditional Chinese Medicine (TCM), China. A total of 292 patients, aged 18 to 60, who complain of subjective tinnitus, will be initially enrolled in our study. The total study period will be 17 to 18 weeks, which includes the following time points: a run-in period of 1 week after screening, a treatment period of 4 weeks and a follow-up period of 12 weeks (Figure 1). The trial is to be executed from January 2014 to December 2014.

### Inclusion criteria

Patients who will be recruited in this study should meet the inclusion criteria including:

1. Present with subjective tinnitus, unilateral or bilateral
2. Be aged between 18 and 60 years old
3. Not be participating any other clinical trials
4. Have no previous acupuncture experience
5. Provide a written informed consent and accept the treatment schedule.

### Exclusion criteria

Patients with any of the following conditions will be excluded:

1. Those with objective tinnitus
2. Those whose history, physical examination and imaging studies reveal any structural etiology for their complaint, such as acoustic tumor, head trauma, and cerebral vascular events
3. Pregnant women or women who are expecting to be pregnant in the recent half year, or lactating women
4. Those who are unwilling to cooperate.

### Withdrawal from the study

The study patients will be allowed or be asked to withdraw from the study if:

1. They would prefer not to be subjected to the assigned treatment for various reasons at any of the trial stages
2. Severe adverse events occur that necessitate their withdraw from the trial
3. They cannot fully participate in the treatment or follow-up.

### Recruitment and randomization procedures

The participants will be recruited through advertisements in local newspapers, hospital websites, and television. Printed recruitment posters will also be posted in the hospital and the university campus. Then, the participants will be randomly assigned to either the manipulation or the non-manipulation group with an assignment ratio of 1:1 according to the random number of allocation sequence generated by computer (using SPSS 19.0 statistical software package, IBM, Chicago, IL, USA). The random allocation numbers are sealed separately in opaque envelopes separately. After the participants have picked-up one of the envelopes, their random numbers and corresponding randomization information, such as the participant’s name in a pinyin format and gender, will be recorded in a random allocation table in duplicate and sealed in an opaque container respectively. The container will not be opened until blinding has ceased. A well-trained acupuncture practitioner, who has practiced for more than ten years, performs the intervention according to the assignment. Also, the acupuncturist does not participate in any of the subsequent evaluation phases. Investigators, including the study physicians and the analyzing statistician, are blinded to the manipulation group assignment.

### Intervention

#### Rational for acupuncture protocol

**Figure 2:**
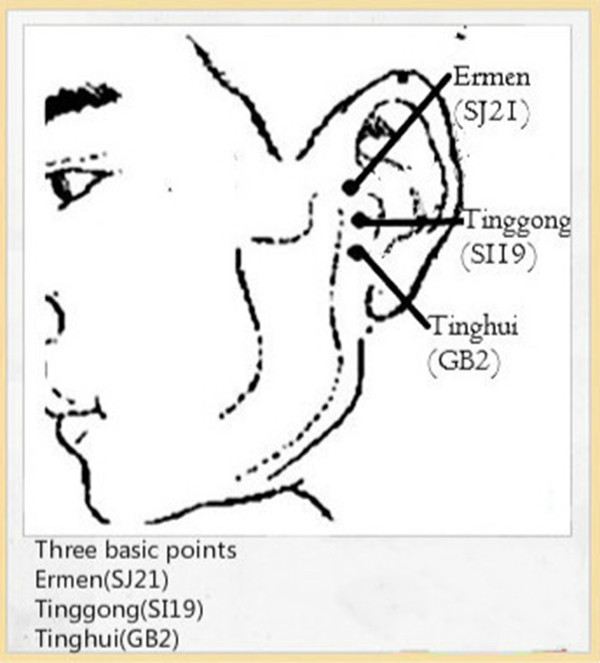
**Location of basic acupoints - three basic points are used for each patient, and include** ***Tinggong*** **(SI19),** ***Ermen*** **(SJ21) and** ***Tinghui*** **(GB2).**

**Figure 3:**
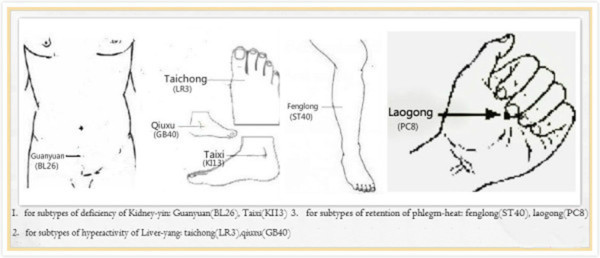
**Location of secondary acupoints - according to syndrome differentiation of tinnitus, the appropriate secondary points could be chosen:** ***Fenglong*** **(ST40) and** ***Laogong*** **(PC8) for retention of phlegm-heat,** ***Taichong*** **(LR3) and** ***Qiuxu*** **(GB40) for hyperactivity of Liver-** ***yang*** **, and** ***Guanyuan*** **(BL26) and** ***Taixi*** **(KI13) for deficiency of Kidney-** ***yin*** **.**

Having screened for a range of acupuncture points for tinnitus through a systematic review of records of ancient books, as well as the standardized acupuncture protocol devised by acupuncture experts in China, we formed the acupuncture protocol for this trial. Three basic points used for each subject are *Tinggong* (SI19), *Ermen* (SJ21) and *Tinghui* (GB2) (Figure 2). These acupoints are on the Small Intestinal Meridian of Hand - *Taiyang*, *Sanjiao* Meridian of Hand - *Shaoyang* and the Gallbladder Meridian of Foot - *Shaoyang*. The three meridians bypass the ear. Because there is a theory stating that therapeutic effects might be elicited by placing needles on acupuncture meridians relating to specific organs, appropriate meridians and acupoints which are in close relationship with the ear are chosen as basic treatment for tinnitus in this study. Also, as has been mentioned in traditional Chinese theory, tinnitus can be classified as three subtypes according to tinnitus-related syndromes: retention of phlegm-heat, hyperactivity of Liver-*yang*, and deficiency of Kidney-*yin*. The first two are excess syndromes while the later belongs to deficiency. Then appropriate secondary acupoints are also added as follows (Figure 3):

1. For subtypes of retention of phlegm-heat: *Fenglong* (ST40), *Laogong* (PC8)
2. For subtypes of hyperactivity of Liver-*yang*: *Taichong* (LR3), *Qiuxu* (GB40)
3. For subtypes of deficiency of Kidney-*yin*: *Guanyuan* (BL26), *Taixi* (KI13)

Participants in either the manipulation group or the non-manipulation group are all diagnosed by the methods of interrogation, inspection, auscultation and palpation. Then, the syndromes of the patients are differentiated in accordance with the clinical materials acquired through the diagnostic methods to classify them into either the syndrome of retention of phlegm-heat, the syndrome of hyperactivity of Liver-*yang*, or the syndrome of deficiency of Kidney-*yin*. Based on the diagnostic pattern of TCM, an appropriate acupuncture plan for each patient is selected individually. We also believe that this selective treatment could be performed more effectively and we hope to explore this in our study.

### Acupuncture administration

All candidates go through a standardized interview and undergo a temporal bone computed tomography (CT) scan, audiological testing of hearing thresholds, minimal masking levels, and loudness discomfort levels. Participants will also be asked to complete the Tinnitus Severity Index Questionnaire (a standard questionnaire with 13 items for quality of life in patients suffering tinnitus), Tinnitus Loudness Questionnaire, the Hospital Anxiety and Depression Scale (HANDS) at the screening visit and at 4, 8 and 16 weeks after randomization. The patients in both the manipulation group and the non-manipulation group are blinded as to which treatment method they would receive. For both groups, treatment consists of five daily sessions of thirty minutes each time for four consecutive weeks.

Acupuncture will be performed by an acupuncturist who has more than ten years experience and has obtained an acupuncture license (Chinese medicine practitioner license), which is licensed by the Ministry of Health of the People’s Republic of China). In order to make the patients comfortable and relaxed during treatment, a constant humidity and a temperature of 20 to 25°C is necessary. Subjects will be advised to assume a supine position on the bed. As has been mentioned above, three basic points and three to four secondary acupoints are selected for each patient according to syndrome differentiation. When the acupoint sites have been located and disinfected with 75% alcohol cotton, a sterile disposable stainless needle of 0.3 mm diameter and 25 mm in length (Suzhou Hualun Medical Appliance Co., Ltd, Jiangsu, China) is inserted gently into the SI19, GB2, SJ21, PC8 and LR3 points separately for a depth of about 0.25 to 0.5 *cun*. For ST40, GB40, BL26 and KI13, a needle with the diameter of 0.3 mm and a length of 40 mm is used with the approximate puncture depth of 0.5 to 1 *cun*. It is advisable to retain the needles for 10 to 20 minutes for the subtypes of deficiency of Kidney-*yin*, while this is not necessary for the other two subtypes with excess syndromes. For the whole treatment, the insertion and withdrawal of the needle is at a constant speed.

For the manipulation group, patients are to be acupunctured with some genuine manipulations by lifting, thrusting, and twirling the needle to generate needle sensation, known as *De Qi* (described above). When *De Qi* occurs, the internal compound sensation of soreness, numbness, distension, dull pain, coldness, warmth, heaviness, and radiation at and around puncture points may be felt. Also, the acupuncturists may also feel the sensation of sinking, stagnancy, tightness and astringency. In non-manipulation group, the acupuncturist inserts the needles without using any acupuncture techniques. Patients receive one treatment session daily for five consecutive days, and the total treatment course lasts for four weeks.

### Assessment of acupuncture sensation ‘*De Qi*’

The Chinese version of the ‘modified’ Massachusetts General Hospital Acupuncture Sensation Scale (C-MMASS) is used to record the patients’ sensations of *De Qi* quantitatively. The C-MMASS was established on the basis of MASS. In C-MMAS, the terms have been translated into precise Chinese, but one description, ‘sharp pain’, was removed from the scale. The validity and reliability of the scale have been examined by exploratory study [15]. The descriptor in C-MMASS includes soreness, aching, deep pressure, heaviness, fullness/distension, tingling, numbness, dull pain, warmth, cold, throbbing, and there is one supplementary row at the end for subjects to describe perceptions in their own words. Each of the elements is presented on a 10-cm bar to indicate the degree of needle sensation from ‘none’, ‘mild’, ‘moderate’ to ‘severe’. Subjects are asked to quantify their sensations at each acupoint immediately after each treatment by rating their intensities on the numerical bar. If the participants have any sensations not given in the scale, they could describe them at the end of the scale. Then, for each subject, the total *De Qi* scores of the 20 sessions will be calculated together for final statistical analysis.

### Measurements of tinnitus

The efficacy of acupuncture treatment for tinnitus is assessed in terms of the severity and loudness of tinnitus. Tinnitus Handicap Inventory (THI) and a 10-cm Visual Analog Scale (VAS) are used. Patients complete the THI and VAS before the treatment and every four weeks during the following period. The 25-item THI consists of three scales: a functional subscale, a catastrophic response subscale and an emotional subscale. Before and after the acupuncture treatment, the participants are asked to report their tinnitus on the THI Questionnaire with ‘Yes’ (4 points), ‘Sometimes’ (2 points) or ‘No’ (0 points). Based on the total THI score, each patient’s tinnitus severity could be categorized into ‘no handicap’ (0 to 16), ‘mild handicap’ (18 to 36), ‘moderate handicap’ (38 to 56) and ‘severe handicap’ (58 to 100).

### Patient safety

Before randomization, patients will be asked to undergo routine tests of blood, urine and stool; an electrocardiogram (ECG), liver function, hepatitis serology, HIV serum antibody assay, blood glucose, and kidney functions are needed to exclude any related serious illness. The results of these tests will allow the assessment of risks associated with catgut implantation at acupoints. If any adverse effect occurs during the trial, patients will be treated as soon as possible.

### Quality control

All acupuncturists are required to receive special training, including the technique for puncture and the way to deal with adverse events. The acupuncturists should also learn how to use the randomization method in order to communicate with patients and to keep them blind to the treatment throughout the trial. Only those who have completed the required training and have passed all necessary examinations will be recruited for this trial. In order to maintain quality of this trial, all outcome assessments will be blinded. In addition, all researchers must understand the purpose and design of this trial. Audits will be conducted regularly on compliance with standard operation procedures every week. Report of audits should be presented to the chief monitor. If patients withdraw from the trial either in the treatment period or in the follow-up phase, the reason should be clarified and the rate should be statistically analyzed.

### Statistical analysis

We conducted a pilot study, in which the similar outcome measurements were used, to determine the sample size prospectively. In this pilot study, the two methods of manipulation and non-manipulation were about 70 and 50% effective respectively when the patients underwent acupuncture 5 times a week for 4 weeks. Based on the population rates of the two groups, the following formula is used to estimate sample size [16]:


Calculations are performed c 5% significance level. In the formula, α = 0.05, Z_0.05_  = 1.96, β = 0.10, Z_0.10_  = 1.282; n_1_, n_2_ represents the sample size of each group respectively and π_1_ = 0.50, π_2_ = 0.70, π_c_ = (π_1_ + π_2_)/2 = 0.60. Thus as a result, an estimated 146 patients per group should be recruited when allowing for a 15% withdrawal rate.

Statistical analysis will be performed using the statistical software package SPSS 19.0 for windows (SPSS Inc, Chicago, IL, USA). Firstly, the baseline characteristics of the two groups, such as age, weight, duration of tinnitus, THI and VAS scores, will be analyzed by an unpaired *t*-test or paired t tests. Then, the second step is to compare the efficacy of *De Qi* or not in the treatment of tinnitus. The average intensity of *De Qi*, the total number of *De Qi* acupoints of all the treatment sessions, the scores of THI and VAS will be tested for normal distribution. If the data follow normal distribution, a two-sample (independent) *t*-test will be used. The data that are not conforming to the normal distribution will undergo Wilcoxon testing. A level of *P*-value less than 0.05 will be considered statistically significant.

Furthermore, the intention-to-treat analysis (ITT) and per-protocol (PP) analysis should be conducted as two principles to test whether the result of this trial is credible. Any cause of withdrawal or loss to follow-up should be clarified. Qualified statisticians will perform the statistical analysis in a blinded manner.

## Discussion

Although acupuncture is one of the major parts of TCM, it is regarded as a useful complementary therapy to conventional medicine and the efficacy of acupuncture has been proven by many randomized controlled trials [17–19]. Also, it has been widely believed that the needle sensation is important in acupuncture treatment. In one of our surveys of acupuncture treatment for tinnitus, most of the patients and acupuncturists believed that stronger needle sensations led to better treatment results. However, it is still believed by some researchers that the therapeutic effects of acupuncture are due to the psychological factors [20]. The relationship between *De Qi* and treatment effects is still controversial. Further studies were conducted to explore the relationship among *De Qi*, psychological factors, and clinical efficacy. Xiong *et al*. [21] reported that it was *De Qi*, but not psychological factors, that determined the therapeutic efficacy of acupuncture treatment for primary dysmenorrhea. Kong *et al*. [22] found significant correlations between analgesia and needle sensations of numbness and soreness on experimental pain, which suggested that attributes of *De Qi* sensations might be useful clinical indicators of effective treatment. As for tinnitus, although there was pilot, sham-controlled and double-blind trial to verify the efficacy of the treatment of acupuncture [18], the degree and the type of *De Qi* were not clarified. Further study should be designed to explore the relationship between *De Qi* and acupuncture points, stimulation and treatment effects.

In a recent systematic review of acupuncture for the treatment of tinnitus [23], a total of nine RCTs were included and *De Qi* was considered in six of these trials. In the present data set, no evidence could be found that *De Qi* exerted an important influence on the clinical outcome. However, there are some flaws in the trials that might influence the quantity and quality of the primary data. First, most of the trials had a high risk of bias, such as lack of power calculation, existing of exclusion or attrition bias due to not reporting the dropouts and withdrawals, and insufficient description of randomization, blinding and allocation concealment. Second, although acupuncture points were selected according to TCM theory in the nine studies, constant acupoints were used for all patients except two [24, 25]. It is more rational to select proper points and treat the patients individually based on differentiation of signs and symptoms of tinnitus. Based on the analysis of the previous studies, some improvements have been made in this study and we hope we will be able to reach a reliable evidence of the efficacy of acupuncture for tinnitus and to know the role of *De Qi* in this treatment.

## Trial status

The recruitment of patients will start 1 June 2014, and it is expected that by 31 October 2014 the required sample size will be reached.

## Abbreviations

C-MMASS: The Chinese version of the ‘modified’ Massachusetts General Hospital Acupuncture Sensation Scale
CT: computed tomography
ECG: electrocardiogram
HANDS: the Hospital Anxiety and Depression Scale
ITT: intention-to-treat analysis
TCM: Traditional Chinese Medicine
THI: the Tinnitus Handicap Inventory
PP: per-protocol
VAS: Visual Analog Scales.
